# Tumour-treating fields (TTFields): Investigations on the mechanism of action by electromagnetic exposure of cells in telophase/cytokinesis

**DOI:** 10.1038/s41598-019-43621-9

**Published:** 2019-05-14

**Authors:** Lukas Berkelmann, Almke Bader, Saba Meshksar, Anne Dierks, Gökce Hatipoglu Majernik, Joachim K. Krauss, Kerstin Schwabe, Dirk Manteuffel, Anaclet Ngezahayo

**Affiliations:** 1 Institute of Microwave and Wireless Systems, Leibniz University Hannover, Hannover, Germany; 2 Institute of Cell Biology and Biophysics, Department of Cell Physiology and Biophysics, Leibniz University Hannover, Hannover, Germany; 3 Department of Neurosurgery, Hannover Medical School, Hannover, Germany; 4 Center for Systems Neuroscience (ZSN), University of Veterinary Medicine Hannover, Foundation, Hannover, Germany

**Keywords:** Cancer therapy, Cell biology

## Abstract

Tumour-treating fields (TTFields) use alternating electric fields which interfere with dividing cells, thereby reducing tumour growth. Previous reports suggest that electrical forces on cell structure proteins interfered with the chromosome separation during mitosis and induced apoptosis. In the present report we evaluate electromagnetic exposure of cells in telophase/cytokinesis in order to further analyse the mechanism of action on cells. We performed numerical electromagnetic simulations to analyse the field distribution in a cell during different mitotic phases. Based thereon, we developed an electric lumped element model of the mitotic cell. Both the electromagnetic simulation and the lumped element model predict a local increase of the specific absorption rate (*SAR*) as a measure of the electromagnetically induced power absorption density at the mitotic furrow which may help to explain the anti-proliferative effect. In accordance with other reports, cell culture experiments confirmed that TTFields reduce the proliferation of different glioma cell lines in a field strength- and frequency-dependent manner. Furthermore, we found an additional dependence on the commutation time of the electrical fields. The report gives new insights into TTFields’ anti-proliferative effect on tumours, which could help to improve future TTFields application systems.

## Introduction

High grade glioma represent the most common and aggressive brain tumour in adults with a median survival after diagnosis of less than one year^1–5^. The standard treatment for newly diagnosed high grade glioma is a surgical resection to the maximal safety possible, followed by radiotherapy and maintenance chemotherapy with temozolomide^6^. More recent advances in surgical and concomitant therapy improved the survival time only to a small extent^1,4,7–9^. Tumour-treating fields (TTFields) represent a relatively new treatment for various tumours including high grade glioma. After it was shown that TTFields improved the progression-free survival and the overall survival, TTFields were approved by the Food and Drug Administration (FDA) for treatment of diagnosed high grade glioma^10,11^. TTFields are used as complement to standard treatment and are even discussed as replacement of chemotherapy^12,13^.

At cellular level, previous studies indicated that TTFields primarily affected mitotic cells^13–16^. It was proposed that proteins with large dipole moments like tubulin dimers would align with the electric field of TTFields, which compromised the mitotic spindle, and thus the mitotic process^14,17–19^. Furthermore, the induction of apoptotic cell death due to strong forces on septin molecules was suggested^20^. Additionally, it was proposed that during the telophase, the electric fields become highly inhomogeneous at the mitotic furrow, so that dielectrophoretic forces influence the biomolecules in the furrow region, subsequently compromising the cell division^12,19^. However, later calculations indicate that a significant impact of TTFields on tubulins and septins is rather unlikely, while dielectrophoretic forces could possibly affect the cellular molecules^21,22^. This shows that to date the exact biophysical mechanisms of TTFields on mitotic cells are not completely understood and more research is necessary in order to optimise the application of TTFields for glioma and other tumour treatment. In the present report we therefore used different modelling approaches to precisely evaluate the impact of TTFields on glioma cells and sought to verify the results of the modelling in cell culture experiments.

Our calculations and experimental data lead to new insights into the effects of TTFields on mitotic cells. Furthermore, the presented models could be helpful to increase the efficiency of TTFields for tumour treatment by finding optimal TTFields parameters.

## Results

The cellular mechanisms by which TTFields repress tumour growth are not clearly understood yet, making a further optimisation of treatment techniques and applications difficult. In this report, power absorption due to the applied electromagnetic fields is investigated by analysing the specific absorption rate (*SAR*), which describes the absorbed power density. The *SAR* is also utilized to define the limits of human exposure to electromagnetic fields^23^ (ICNIRP, FCC etc.) without reaching excessive (e.g. damaging) tissue heating. It can be directly calculated from the electromagnetic fields as follows^24^:

1 $$SAR=\frac{{\rm{\sigma }}\cdot |E{|}^{2}}{\rho }({\rm{local}}\,{\rm{SAR}})$$

With *E* representing the electric field strength (V_rms_/m) in the tissue, σ gives the electric conductivity (S/m) and ρ is the volumetric mass density (kg/m^3^).

With the assumption of non-thermodynamic circumstances, e.g. no thermal diffusion etc., the *SAR* would be directly related to the increase in temperature as given by the equation:

2 $$SAR={\frac{c{\rm{\Delta }}T}{{\rm{\Delta }}t}|}_{t=0}$$

With Δ*T* representing the temperature increase (K), Δ*t* the duration of exposure (s) and *c* the specific heat capacity (J ⋅ kg^1^ ⋅ K^−1^).

However, since the thermodynamic circumstances usually are more complicated, often only the *SAR* is calculated as mean value over a volume of tissue, e.g. 10 g in ICNIRP guidelines^23^, and used as the measure for potential temperature increments induced by electromagnetic fields. Even if the *SAR* is meant to describe thermal effects it can also be utilized as general measure for all power-dependent effects induced by electromagnetic fields.

**Figure 1:**
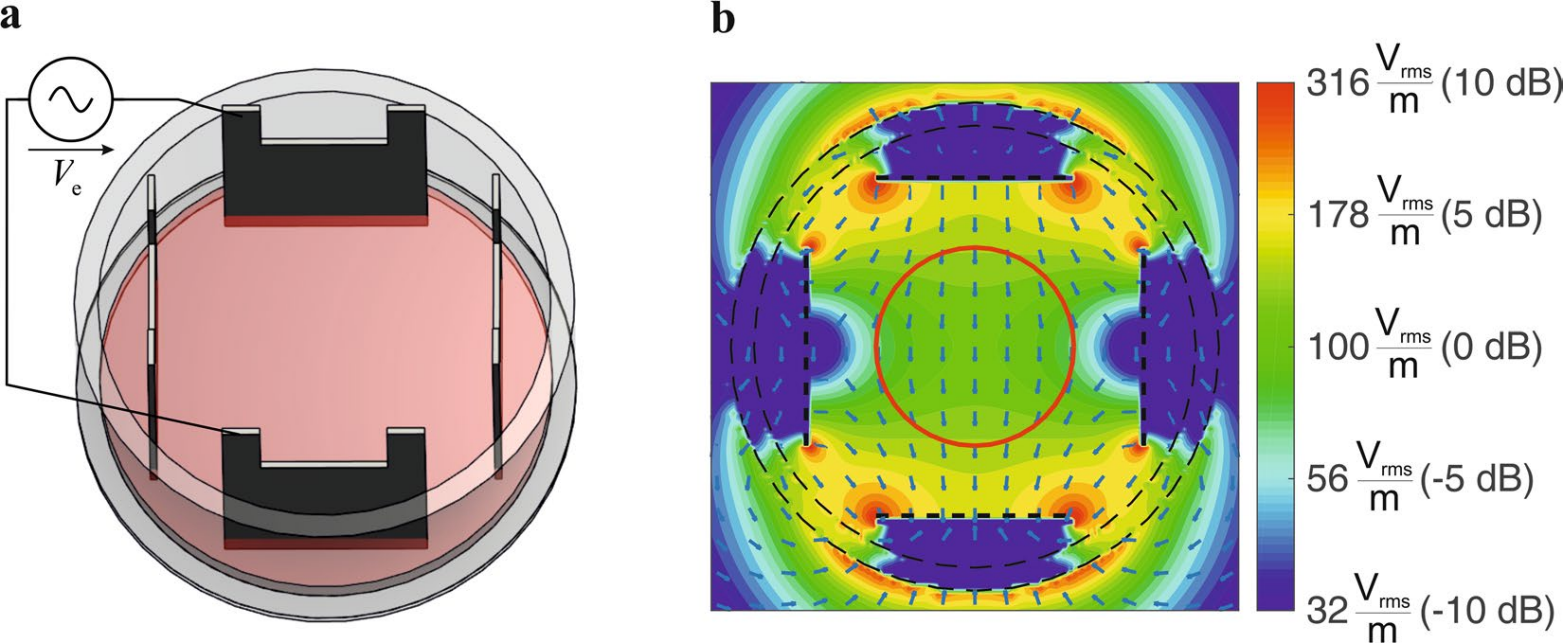
(**a**) Electrode setup for *in-vitro* TTFields exposure system. (**b**) Simulated electric fields in utilized setup, applied voltage *V*_*e*_ = 10 V_pp_. Cells were cultivated within the area delineated by the red circle in the middle of the setup (*d* = 15 mm).

In the first step we performed electromagnetic simulations on the field distribution in the developed exposure setup shown in Fig. 1 (details on the setup and simulations are presented in the materials and methods section). For the culture media, a conductivity σ = 1.3 S/m was determined by measurements and a relative permittivity ε_r_ = 80 and a volumetric mass density of ρ = 1000 kg/m^3^ was assumed. As in the considered frequency range inside the culture media conduction currents far exceed displacement currents ($\sigma \gg \omega {\epsilon }$) the exact permittivity value is not necessary.

The results of the simulations reveal an almost homogeneous electric field distribution in a circular region with a diameter of *d* = 15 mm near the middle of the cell dish. Therefore, cells were cultured primarily in this region (Fig. 1b, red marked area). Simulations of the exposure setup also show that since the problem fulfils the conditions for quasistatic approximations, the applied electromagnetic fields affect the media almost independently of the frequency. However, as expected from equation 1, while the field strength increases in direct proportion to the increase of the applied voltage, the increase in *SAR* is proportional to the square of the applied field strength (Table 1). To analyse the heating effect of TTFields on the culture medium, we continuously recorded the temperature in the culture media during application of TTFields with different settings. It was shown that the temperature only increases slightly in the TTFields settings used in the present report (*V*_*e*_ ≤ 12 *V*_*pp*_), indicating that a sole effect on the cell culture medium is rather unlikely (Table 1). Further, we analysed whether TTFields could affect cellular growth by changing the properties of the culture medium. However, we found that medium pre-treated by TTFields for 72 h did not significantly affect the cell proliferation within a cultivation period of 72 h (data not shown).

**Table 1:** Calculated electric field strengths *E* and *SAR* as well as the measured temperature increase *dT* (steady state) in the culture medium in response to applied voltages *V*_*e*_ at *f* = 100 kHz.

*V*_*e*_	5 V_pp_	7 V_pp_	9 V_pp_	12 V_pp_	15 V_pp_
*E* in V_rms_/*m*^a^	57.4 ± 7.3	80.4 ± 10.2	103.3 ± 13.1	137.8 ± 17.4	172.2 ± 21.8
*SAR* in W/kg^a^	4.4 ± 1.1	8.5 ± 2.1	14.1 ± 3.5	25.1 ± 6.1	39.15 ± 9.6
*dT* in K^b^	0	0.2	0.4	0.7	1.1

^a^Mean value ± SD, averaged over the area with a diameter *d* = 15 mm containing the cells (Fig. 1).

^b^The temperature was recorded in the centre of the cell culture dishes.

**Figure 2:**
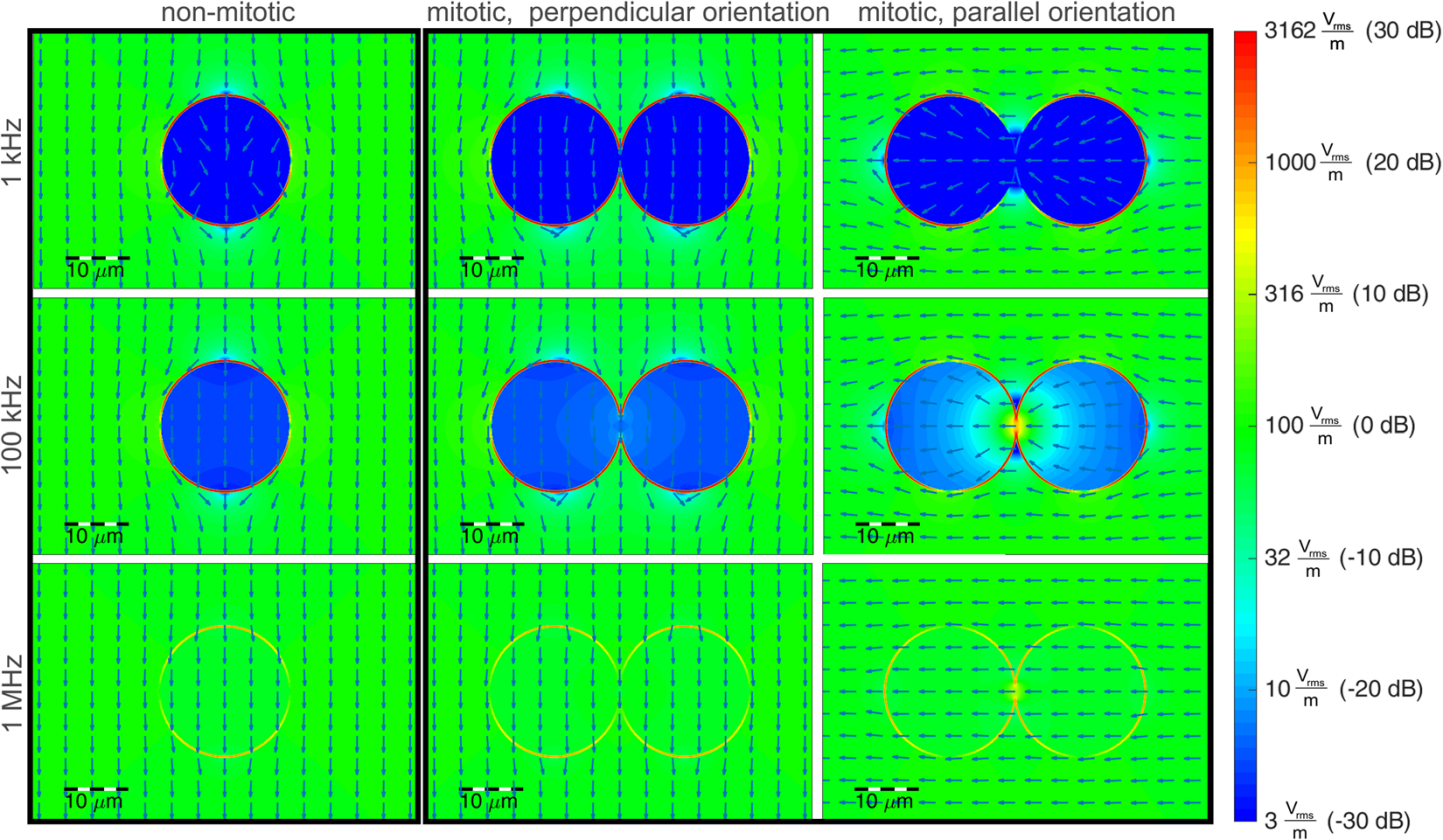
Calculated electric field distribution by application of a homogenous electrical field of *E* = 100 V_rms_/m. Assumed parameters: cell diameter *d*_*c*_ = 20 μm, membrane thickness and permittivity chosen to realize a capacitance of *C*_*m*,*ges*_ = 29 pF, relative permittivity of external medium and cytosol: *ε*_*r*_ = 80, conductivity  *σ* = 1.3 S/m. For the logarithmic (colour) scaling in dB we calculated $\frac{E}{{E}_{0}}$, with *E*_0_ the mean field strength in the surrounding medium.

Therefore, in the second step, we performed electromagnetic simulations at cellular level. Since it was described that TTFields affect cells in mitosis, we considered cells in mitosis, which are almost spherical. The cell (exemplary for the glioma cell line BT4Ca) was modelled as a sphere with a diameter *d*_c_ = 20 µm (according to a mean measured cell volume of 3.69 pl). The cells in telophase/cytokinesis (immediately before separation of the daughter cells) were modelled with a mitotic furrow with a diameter *d*_f_ = 2 µm. Furthermore, the material parameters of the intracellular medium were assumed to be nearly the same as for the extracellular culture medium (relative permittivity *ε*_*r*_ = 80 and conductivity *σ* = 1.3 S/m). A membrane capacitance *C*_*m,tot*_ = 29 pF and a membrane resistance *R*_*m,tot*_ = 600 MΩ were measured using patch-clamp technique. Figure 2 depicts the results of our simulations at cell level in the form of the electric fields normalised to the field strength in the surrounding medium.

**Figure 3:**
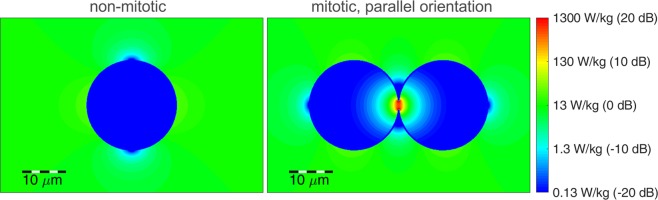
Calculated local SAR in response to TTFields (*E* = 100 V_rms_/m) applied at *f* = 100 kHz (s. Fig. 1). For the logarithmic scaling in dB we calculated $\frac{SAR}{SA{R}_{0}}$, with *SAR*_0_ the mean *SAR* in the surrounding medium.

For a cell not in telophase/cytokinesis (Fig. 2, first column), the emerging effects depend only on a well-known mechanism^25^: At low frequencies the cell membrane effectively shields the inner cell from the electric fields, due to the high resistance compared to the external culture medium and the cytosol. Meanwhile, at higher frequencies the membrane’s capacitance provides a parallel conducting path for displacement currents, which increase with frequency and begin to shorten the membrane’s resistance at around 1 MHz. However, for cells in telophase/cytokinesis the electromagnetic simulations of the field distribution (Fig. 2, second/third column) show excessive electric fields in the cleavage furrow region for frequencies around 100 kHz. This obviously only takes place if the electric field polarisation is parallel to the longitudinal axis of the cells hourglass shape. Similar results were already published by other authors^17,22,26^. Furthermore, we evaluated the resulting *SAR* in order to investigate effects caused by the electromagnetic fields at cell level. Figure 3 depicts the local *SAR* distribution resulting by TTFields application at a frequency *f* = 100 kHz. The *SAR* calculated for cells not in telophase/cytokinesis and for cells in telophase/cytokinesis is normalised to the *SAR* in the surrounding medium. It can be observed that the local *SAR* inside the cleavage furrow regions exceeds the value of the surrounding medium by a factor of approximately 17.6 dB, which gives a power absorption density in this region of about 57 times higher (Fig. 3).

**Figure 4:**
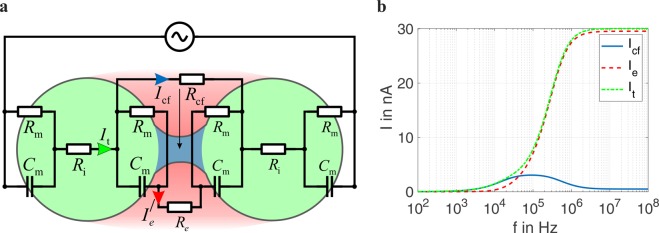
(**a**) Schematic representation of the lumped element model of a cell in telophase/cytokinesis exposed to an electric field polarised parallel to the longitudinal axis of the hourglass shape of the cell. See methods section for used element parameters. (**b**) Frequency-dependent currents in the cleavage furrow region as calculated with the lumped element model.

To investigate other parameters by which TTFields affect the cells, e.g. the frequency of the applied electric field, we developed a lumped element circuit representation to model the cells’ electromagnetic behaviour during mitosis (Fig. 4a). A similar model for single cells was already utilized by other authors^27^. Based thereon, we extended the equivalent circuit to model cells in the telophase/cytokinesis stage. The electrical lumped element parameters (capacitance and resistance values) were chosen according to the geometries and electromagnetic material parameters as assumed in the numerical EM simulation. The currents calculated in the lumped element model reveal the same overall trends found from the electromagnetic field simulations (Fig. 4b). Considering the total current *I*_t_ flowing through a cell not in telophase/cytokinesis, we found, as reported before, that at low frequencies the inner cell is shielded by the high impedance of the cell membrane which almost suppresses the current^22,28^. In the frequency range of tenths of kilohertz, the membrane capacity *C*_m_ begins to shorten the membrane resistances *R*_m_ (Fig. 4).

For cells in telophase/cytokinesis, there are two possible paths for the currents: one leading through the narrow cleavage furrow (*I*_cf_, blue marked region), the other through the membranes and partly through the extracellular medium (*I*_e_, red marked region). The calculation from the lumped element model reveals the same strongly frequency-dependent effect of the current flowing through the cleavage furrow region *I*_cf_. The furrow current *I*_cf_ reaches a maximum at frequencies which are close to the optimal frequencies found in former studies for maximizing the anti-proliferative effect of TTFields.

**Figure 5:**
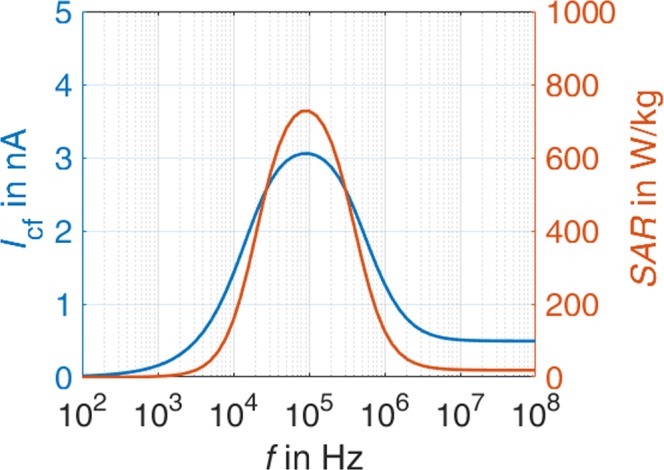
Simulated *SAR* in the cleavage furrow region.

Assuming a uniformly distributed current in the cleavage furrow region we also calculated the local *SAR* from the lumped element model (Fig. 5). Because of the proportional relation between *SAR* and the square of the current (*SAR*~*I*^2^), the frequency range showing excessive *SAR* values is narrower compared to the frequency range showing excessive current values. The effect of excessive power absorption only takes place in cells with a narrow mitotic furrow orientated parallel to the fields. Because of the random furrow orientation, the field polarisation should change periodically as also assumed in earlier studies^17,18,26,29^.

**Figure 6:**
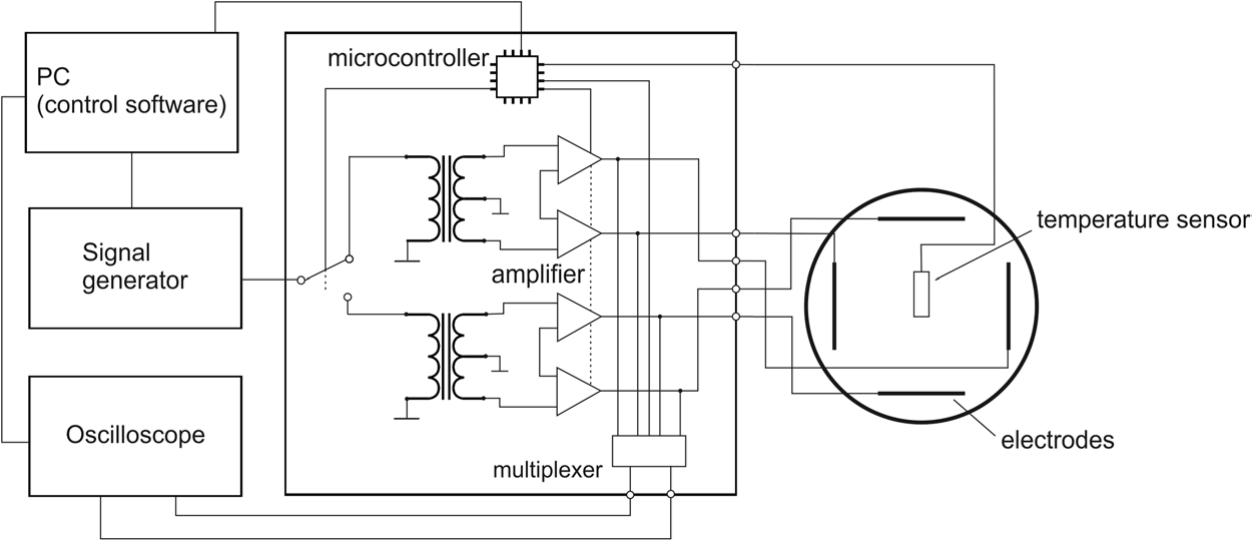
Block diagram of the developed TTFields exposure setup.

To verify the modelled parameters, we cultivated four different rat glioma cell lines (BT4Ca, C6, F98, RG-2) and applied TTFields at different field strengths, frequencies, and commutation times using our experimental setup (Fig. 6). As shown in Table 2, the application of TTFields (*V*_*e*_ = 5 to 12 V_pp_, *f* = 200 kHz) reduced the proliferation of the cell lines included in the study in a field strength-dependent manner. The effect is observed for all four cells lines after an application time of 72 h. Moreover, 72 h was the maximal cultivation time during which growth of the control cells could be sustained without renewal of the cultivation media. Therefore, we chose an application time of 72 h for further analyses of the effect of TTFields on the cells.

**Table 2:** Cell numbers of different glioma cell lines counted after TTFields application (*f* = 200 kHz) for 72 h. The counted cell numbers were normalised to the untreated control cells (cont.). The cell numbers (%) are given as average ± SEM from at least three replicates.

Cell line	cont.	5 V_pp_^a^	7 V_pp_^a^	9 V_pp_^a^	12 V_pp_^a^
BT4Ca	100.0 ± 5.3	96.8 ± 2.9	90.7 ± 0.9	73.1 ± 4.6	76.1 ± 8.5
C6	100.0 ± 5.2	108.6 ± 5.0	106.2 ± 12.7	70.9 ± 4.1	77.8 ± 2.2
F98	100.0 ± 11.8	101.9 ± 11.6	80.8 ± 21.2	80.2 ± 12.6	72.3 ± 7.0
RG-2	100.0 ± 5.5	102.8 ± 5.3	93.5 ± 1.6	98.1 ± 11.4	48.1 ± 3.3

^a^See Table 1 for corresponding field strength.

**Figure 7:**
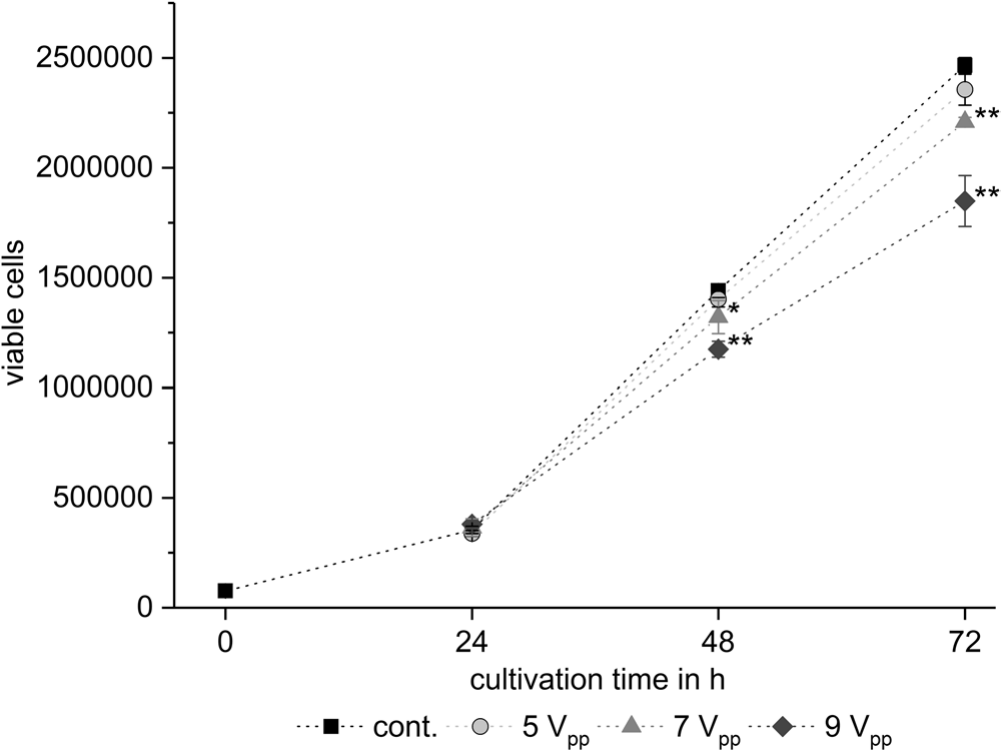
Time- and field strength-dependent reduction of BT4Ca cell numbers after application of TTFields. Average cell numbers ± SEM from at least three replicates are shown. Significant differences compared to the control (cont., Student’s *t* test) are marked with asterisks: *P < 0.05, **P < 0.01.

The reduction of the cell number depends on the duration, intensity, and frequency of the applied TTFields. As exemplary shown for BT4Ca cells, application of TTFields for 24 h, 48 h and 72 h with a voltage of *V*_*e*_ = 5 to 9 V_pp_ gradually reduced the cell numbers (Fig. 7). While application of 5 V_pp_ did not affect the cell proliferation, application of 7 V_pp_ for 72 h reduced the cell population by approximately 10% as compared to the control. The increase of the voltage to 9 V_pp_ enhanced the anti-proliferative effect of TTFields to nearly 30% (Fig. 7). Similar results were obtained for the other cell lines (Table 2).

**Figure 8:**
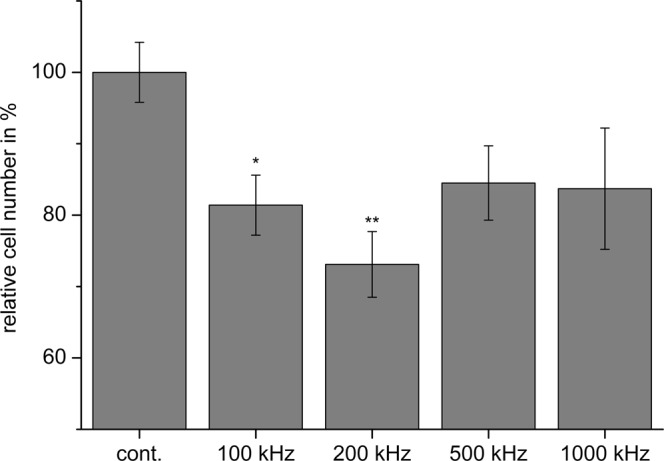
Frequency-dependent reduction of BT4Ca cell numbers after application of TTFields (9 V_pp_) for 72 h. Average cell numbers (% of control (cont.)) ± SEM from at least three replicates are shown. Significant differences compared to the control (Student’s *t* test) are marked with asterisks: *P < 0.05, **P < 0.01.

With respect to the frequency, the cell counting studies reveal a frequency-dependent efficiency of the TTFields. Applying *V*_*e*_ = 9* V*_*pp*_ at 100 kHz, 200 kHz, 500 kHz, and 1000 kHz, we found that only TTFields application at frequencies in the range of 100 kHz to 200 kHz significantly reduced the cell proliferation, while higher frequencies did not. As shown for BT4Ca cells, a reduction of the cell number by about 20% to 30% was achieved at 100 kHz and 200 kHz, while 500 kHz and 1000 kHz only had a mild, non-significant effect on the reduction of cell numbers (Fig. 8). Similar results are observed for other cell lines used in the report (Table 3). These data correlate with the prediction of the lumped element model, that only in a particular frequency range high *SAR* values arise in the mitotic furrow (Fig. 4).

**Table 3:** Cell parameters and cell proliferation after application of TTFields (*V*_*e*_ = 9 V_pp_) for 72 h at different frequencies. The counted cell number for each frequency was normalised to the cell number in controls (cont.) when TTFields was not applied. The cell numbers (%) are given as average ± SEM from at least three replicates for each frequency.

Cell line	Doubling time [h]	Diameter [µm]	cont.	100 kHz	200 kHz	500 kHz
BT4Ca	14	17.1 ± 0.2	100.0 ± 4.2	81.4 ± 4.2	73.1 ± 4.6	84.5 ± 5.2
C6	6–14	12.7 ± 0.3	100.0 ± 3.8	103.1 ± 6.7	70.9 ± 4.1	92.5 ± 8.5
F98	16–30	14.6 ± 0.3	100.0 ± 10.0	85.1 ± 9.0	80.2 ± 12.6	110.5 ± 26.8
RG-2	7–15	14.2 ± 0.1	100.0 ± 15.8	88.4 ± 18.5	98.1 ± 11.4	84.7 ± 7.1

**Figure 9:**
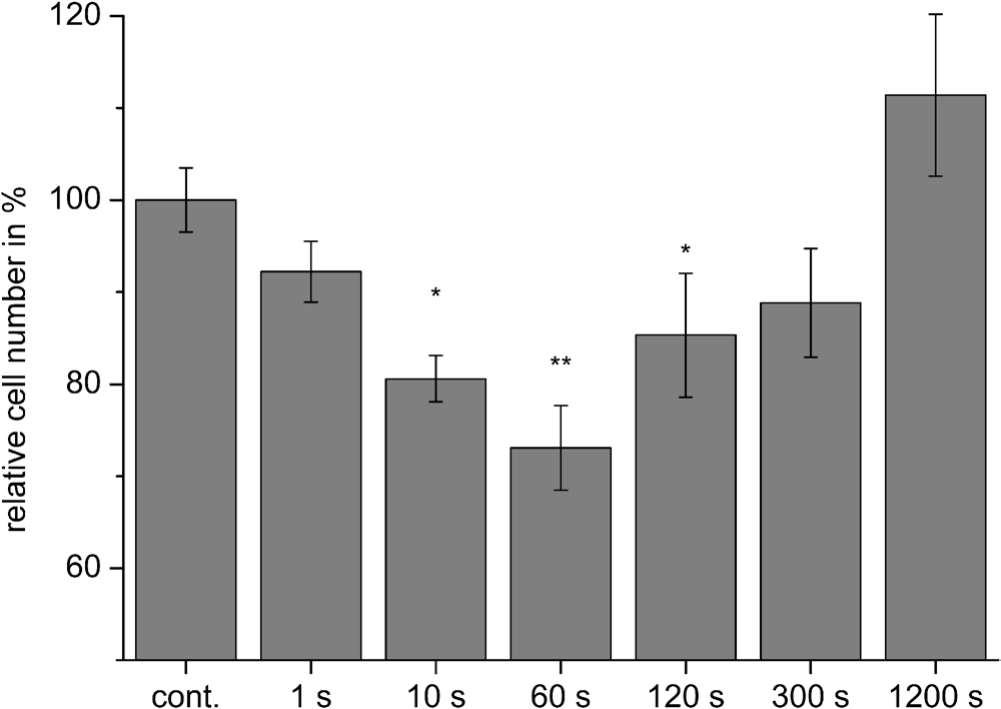
Commutation time-dependent reduction of BT4Ca cell numbers after application of TTFields (9 V_pp_) for 72 h. Average cell numbers (% of control (cont.)) ± SEM from at least three replicates are shown. Significant differences compared to the control (Student’s *t* test) are marked with asterisks: *P < 0.05, **P < 0.01.

With respect to the commutation time, in the experimental setup different commutation times (1 s, 10 s, 30 s, 60 s, 120 s, 300 s, and 1200 s) were tested. For BT4Ca cells, a rapid commutation time of 1 s did not significantly affect the proliferation of the cells (Fig. 8). An increase in the commutation time to 10 s increased the anti-proliferative effect of the TTFields, resulting in a reduction of BT4Ca cell numbers of about 20% after 72 h. This effect was maximal at a commutation time of 60 s, resulting in nearly 30% less BT4Ca cells after an application time of 72 h. For a commutation time of 120 s, a reduction of 20% of cells was observed, while longer commutation times again did not significantly affect the cell numbers (Fig. 9).

**Figure 10:**
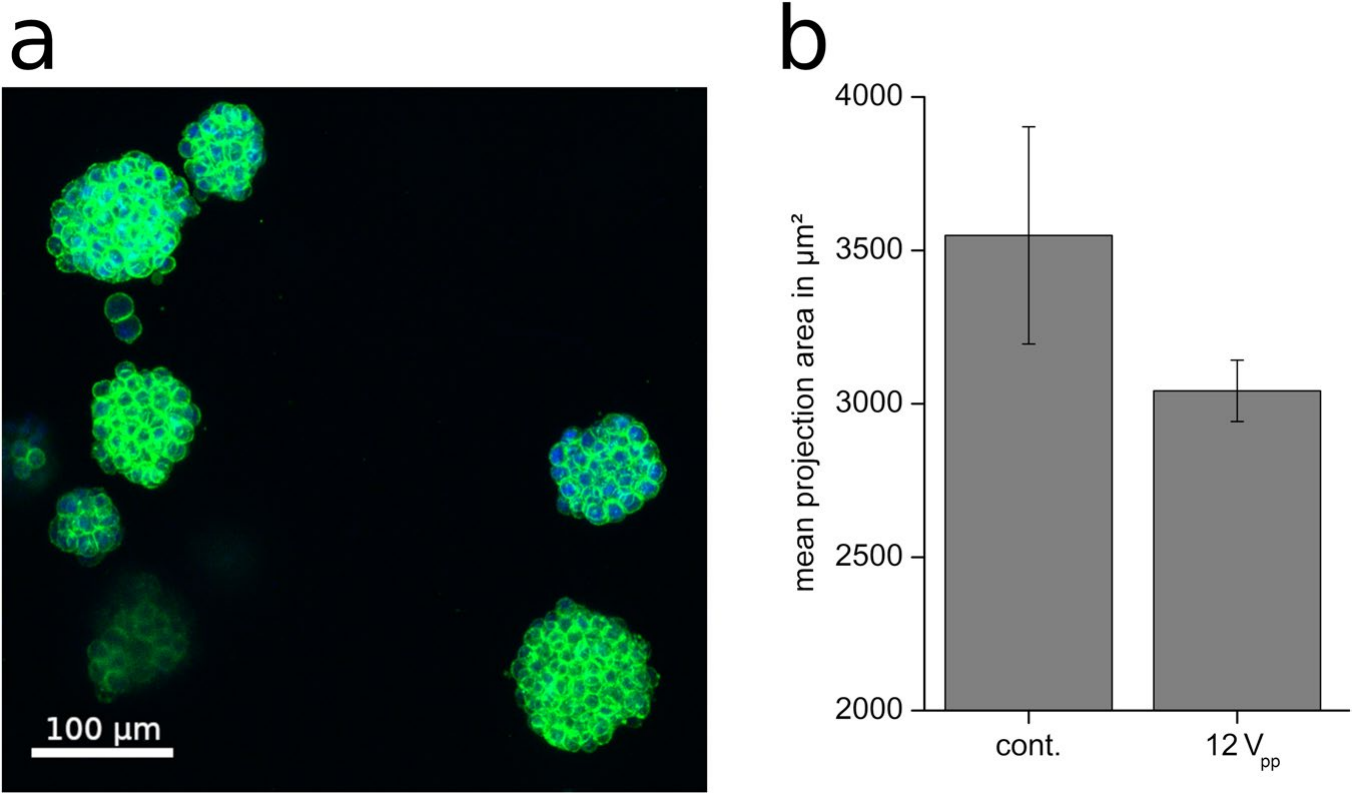
(**a**) Representative images of BT4Ca cells cultured in collagen I gels and stained for actin filaments (green) and nuclei (blue). (**b**) Reduction of the mean projection area of BT4Ca cell clusters cultured three-dimensionally in collagen I gels after application of TTFields (12 V_pp_) for 72 h. Results are given as average ± SEM from five replicates.

*In vivo* tumours grow three-dimensionally, surrounded by an extracellular matrix. Therefore, we tested whether TTFields exerted an anti-proliferative effect on cells in three-dimensional structures. As shown in Fig. 10a BT4Ca cells cultured in a collagen matrix form globular entities, whose size increased with the cultivation time. Application of TTFields (*V*_*e*_ = 12 V_pp_; *f* = 200 kHz) for 72 h and with a commutation time of 60 s resulted in a trend to reduce the growth of the entities, reducing the mean projection area of the spheroid cell clusters by about 15% (Fig. 10b). This result suggests that TTFields may also affect cells in tumour-like three-dimensional structure and that our application setup is applicable to further test the effect of TTFields on three-dimensionally cultured cells.

## Discussion

Tumour-treating fields (TTFields) represent a new clinically applied therapeutic method for various tumours, including high grade glioma. Clinical data showed that TTFields reduce tumour growth, prolonging thereby the life of patients^10–13^. In the present report we show that TTFields reduce the proliferation of glioma cells (Table 2, Fig. 7) without completely stopping it, which might explain why, applied in clinical studies, TTFields till now can not completely stop but only slow tumour growth^13,30^. Concerning the effects of TTFields on cellular level, previous studies have pointed out a susceptibility of mitotic cells to TTFields^17,18,26,29–32^. It was argued that TTFields affected the formation of the mitotic spindle and thereby induced cell cycle arrest and eventually apoptosis in the cells^17,18,26^. Since TTFields did not completely stop the cell proliferation in our experiments (Fig. 7), we assume that TTFields do not affect all, but only a specific portion of mitotic cells.

We used a simplified TTFields application setup (Fig. 1) that was designed to generate an almost homogenous electrical field distribution in the region where the cells are cultivated. Based thereon, simulations were performed on the electric field distribution at the cells in different stages of mitosis. An excitation frequency of *f* = 100 kHz revealed highly inhomogeneous fields during the telophase/cytokinesis stage (Fig. 2). Earlier publications have presented similar results^17,18,26,29,31,33^. However, considering also the power absorption induced by the electromagnetic fields we found that in the mitotic furrow region of cells in the telophase/cytokinesis stage the *SAR* was increased compared to the cells environment by approximately 17.6 dB, leading to an *SAR* approximately 57 times higher than in the cultivation milieu. According to our studies, the field strengths that induce antimitotic effects are around 100 V_rms_/m (Tables 1, 2). Our temperature recordings revealed that in our setup this field strength setting is right below the threshold of inducing an overall temperature rise in the culture medium (temperature rise: *dT* < 0.5 K, Table 1). However, due to the strongly increased power absorption induced by the electromagnetic fields inside the mitotic furrow substantial local effects seem likely. The findings also suggest that only cells in the telophase/cytokinesis stage with the narrow mitotic furrow are sensitive to TTFields since we found a large increase of *SAR* in the furrow region (Fig. 3). In general, the orientation of mitotic cells is statistically distributed. The simulations showed that the power absorption decreases when the furrow is not parallel to the field (Fig. 2, right column). This may explain why, as shown in Fig. 7 and in Table 2, TTFields only reduced, but not completely stopped cell proliferation. Moreover, it can also explain why the application of TTFields in cells cultivated in a collagen matrix with more possible spatial orientations of the mitotic furrow to the field was less effective to reduce the volume increase of the cell spheres when applied for only 72 h (Fig. 10). Although the collagen matrix alone did not affect the conductivity of the extracellular medium we do not know how the matrix may affect the TTFields, which could also participate in reducing TTFields effectivity. However, data presented in Fig. 10 show that the TTFields affected the increase in the projection area of the cell spheres which directly correlates with the sphere volume. In different imaging experiments we found that the increase of the cell sphere volume correlates with an increase of cell numbers within the sphere. A release of the cells from the matrix for cell counting is difficult, therefore we do not know the proportionality between the increase of the sphere volume and that of the cell population. An under- or overestimation of the data presented in this report cannot be excluded. Additionally, for three-dimensionally cultivated cells in a matrix, a better characterization of the matrix and its interaction with TTFields is still needed. Likewise, a specifically designed TTFields application device that allows an application in different spatial directions could increase the efficiency of TTFields.

The electromagnetic simulations and the additionally developed lumped element model of mitotic cells showed that the power absorption in the mitotic furrow region is frequency-dependent (Fig. 5) and therefore allow a qualitative prediction of the experimental parameters. However, some discrepancies between the cell behaviour should be noted. The maximal reduction of the proliferation was not achieved at the same TTFields amplitude and frequency (Tables 2, Fig. 5). It is possible that combined cell properties such as the dimension and the doubling time may have an impact on the results. Further considering variable cell diameters, and thus variabilities in the membrane capacitance, and recently published data that show variability in the conductivity of the cytoplasmic cytosol^34,35^, a modelling of the *SAR* would be changed to about 450 W/kg and 1100 W/kg by a conductivity of the cytoplasmic cytosol of 0.8 S/m and 2 S/m, respectively. Likewise, varying the capacitance of the cell membrane from 10 pF to 30 pF shifts the frequency of maximal *SAR* from 200 kHz to 60 kHz. A further remarkable result of the report is the finding that the anti-proliferative effect is affected by the commutation time. Accordingly, the maximal reduction of the cell proliferation was observed when we applied 9 to 12 V_pp_ at 200 kHz with a commutation time of 60 s (Table 2; Figs 8, 9). A very fast commutation time of 1 s did not significantly reduce the cell numbers (Fig. 9). Considering the duration of mitosis, live cell imaging confirmed that the telophase/cytokinesis stage in BT4Ca cells lasts for approximately 2–4 min. Therefore, a rapid commutation time (<10 s) does not seem to be enough to perturb the completion of mitosis. On the other hand, longer commutation times (>5 min) would probably enable more cells with their mitotic furrows not parallel to the electrical field (Fig. 4) to complete mitosis before being affected by TTFields and thereby could reduce the overall efficiency of TTFields.

As an outlook, our findings on the mechanism of action of TTFields may also have the potential for further improvements of TTFields application systems. For example, further analysis of the influence of the commutation time on the anti-proliferative effect could enhance the TTFields efficacy. Furthermore, our equivalent circuit model may be used to give a first reliable approximation of optimal application frequencies for various kinds of tumour cells as the circuit element parameters are directly linked to the dimensions and material parameters of the cell. However, since cell properties such as the membrane capacitance or cytosol conductivity may vary, the application setup should be conceived with the possibility of experimental fine tuning of the frequency and the amplitude of the applied TTFields.

## Methods

### TTFields exposure setup

We developed an exposure setup that allows an accurate adjustment of the electrical fields in a cell culture system and thereby the investigation of possible interactions between the cells and the electrical fields. At the same time, the system allows the application of small voltages (less than 20 V) to reach the desired field strengths. For exposing the cell cultures to homogenous fields we utilized a setup of four stainless steel electrode plates dipped into to cell culture medium (Fig. 1). Two of the four electrodes were excited at once, so the polarisation of the electric field could be oriented in two different directions. Signals were generated through the function generator DG1022Z (Rigol Technologies, Beijing, China). A custom-designed circuitry was used to amplify and to variably distribute the signals to the electrodes (Fig. 6). To monitor and regulate the voltages at the electrodes we utilized an oscilloscope DS1102E (Rigol Technologies), its input channels could be multiplexed by our custom circuit design to measure all four electrode voltages sequentially. The whole setup was controlled through a PC by a MATLAB (MathWorks, Natick, USA) routine which evaluated the voltages measured at the electrodes and regulated the signals amplitude of the function generator. Furthermore, we integrated a digital temperature sensor (Maxim Integrated DS18B20, San Jose, USA) at the electrodes to monitor the temperature of the cell medium during TTFields application.

### Electromagnetic simulations

Numerical electromagnetic simulations were performed using the quasi-static EM solver of Sim4Life (ZMT Zurich MedTech AG, Switzerland, www.zurichmedtech.com)^36^. For our calculation, the membrane thickness and its permittivity were chosen to emulate the measured membrane capacitance. To verify our calculation we utilized mie series calculations, an analytical solution for scattering of plane waves at multilayer spheres^37,38^. The lumped element model in Fig. 4a represents an extended version of the electromagnetic cell model presented by Ellappan & Sundararajan^27^ to model cells in telophase/cytokinesis stage. Table 4 summarizes the lumped element values that were used in our calculations. These were chosen according to the cells geometry and material parameters to represent the electromagnetic behaviour of the cell model as in the numerical simulations (Fig. 2).

The conductivity of the used culture medium was determined at a frequency *f* = 1 MHz by measurements with an Agilent E4991A RF Impedance/Material Analyser (Agilent Technologies, Santa Clara, USA) and the N1501A Dielectric Probe Kit (Agilent Technologies, Santa Clara, USA). Since theoretically ionic solutions exhibit no dielectric dispersion at frequencies below 1 MHz^39^ the measured conductivity should also be valid in the frequency range utilized in this contribution. To validate this assumption we also compared the impedance between two opposite electrodes of our developed TTFields exposure setup with values obtained by numerical simulations setting the conductivity to the measured value. Measurements of the impedance in our TTFields exposure setup were made with an Agilent Precision LCR Meter 4284 A (Agilent Technologies, Santa Clara, USA) at f = 100 kHz. The impedance obtained in the simulation is in good agreement with the measured value. As in the considered frequency range inside the culture media conduction currents far exceed displacement current (σ ≫ ωϵ) the exact permittivity value is not necessary.

### Cell culture

BT4Ca (Institute of Cell Biology, Department of Cancer Research, University of Essen Medical School, Germany), C6, F98 and RG-2 cells (Uniklinikum Erlangen, Neuro-oncological Research Laboratory) were cultured in tissue culture dishes (Sarstedt AG & Co, Nümbrecht, Germany) in Dulbecco’s modified Eagle’s medium (DMEM, FG 0445, Biochrom GmbH, Berlin, Germany), supplemented with 10% heat-inactivated foetal calf serum (Biochrom GmbH), 1 mg/ml penicillin and 0.1 mg/ml streptomycin (Biochrom GmbH) and 1 x non-essential amino acids (Biochrom GmbH). Cells were kept in an incubator at 37 °C with 5% CO2 and split once to twice a week at 80–90% confluence.

### Whole-cell patch-clamp analysis

Estimation of the membrane capacitance and membrane resistance was performed under whole-cell configuration of the patch-clamp method^40^ at room temperature using the EPC 10 USB double patch-clamp amplifier and the software PatchMaster (HEKA Elektronik Dr. Schulze GmbH, Lambrecht/Pfalz, Germany). Cells grown on glass cover slips were placed in a perfusion chamber containing 0.5 ml of a bath solution composed of (in mM): 145 NaCl, 5 KCl, 2 CaCl_2_, 1 MgCl_2_, 10 glucose, and 10 HEPES, (pH 7.4, 295 mosmol/l). The perfusion chamber was mounted onto an inverted microscope (Zeiss, Oberkochen, Germany). The cells were washed with 20 ml of bath solution at 5 ml/s. To visualise the cells, a CCD camera coupled to the software Aquacosmos (C4742-95, Hamamatsu Photonics K.K., Hamamatsu, Japan) was used. To navigate the patch-clamp capillaries onto the cells, a MicroStar micromanipulator (Scientifica, East Sussex, U. K.) was used. The patch-clamp capillaries were filled with a pipette solution that contained (in mM): 135 K-gluconat, 5 KCl, 0.5 Na_2_ATP, 2.5 MgATP, 0.5 CaCl_2_, 5 EGTA, 5 glucose, and 10 HEPES (pH 7.4, 295 mosmol/l). After establishing a giga-seal, a routine to compensate the capillary capacitance was run and a whole-cell configuration was established. Thereafter a routine to compensate the cell-capacitance was run. The cell capacitance as well as the resistance of the cell membrane was directly read from the control panel in the software PatchMaster.

### Application of TTFields

For experiments 45 000 cells were seeded into the middle of each well of tissue culture 6 multiwell plates (TPP Techno Plastic Products AG, Trasadingen, Switzerland), so that the cells only grew in a distinct area with a diameter of about 15 mm in the middle of the wells in which homogeneous electrical fields were expected (Fig. 1). After 24 h TTFields were started with different voltage, frequency and commutation time settings. For counting the cells were washed with PBS + EDTA (137 mM NaCl, 2.8 mM KCl, 10 mM Na_2_HPO_4_, 1.8 mM KH_2_PO_4_, 3.4 mM EDTA; pH 7.4, 295 mosmol/l). The cells were then trypsinised by adding 250 µl trypsin solution (0.25% in PBS + EDTA, Sigma-Aldrich, Taufkirchen, Germany) per well for 3–5 min. Then 500 µl cell culture medium was added to each well. Cell samples were diluted in CASYton (OLS OMNI Life Science GmbH & Co. KG, Bremen, Germany) and the cells were counted with a CASY TT cell counter (OLS OMNI Life Science GmbH & Co. KG). The number of viable cells per well was averaged from at least three independent biological replicates. Paired Student’s *t* tests between cell numbers from untreated control samples and after TTFields application were performed for statistical analysis. The CASY TT cell counter was also used to estimate the diameter and the doubling time of the cells.

For three-dimensional cell culture 25 000 BT4Ca cells in 68 µl culture medium were mixed with 32 µl rat collagen I solution (5 mg/ml, Trevigen, Gaithersburg, USA) to give a final concentration of 1.6 mg/ml collagen I. This mixture was pipetted per well of 6 multiwell plates and solidified in the incubator for 20–30 min. Then 1.5 ml cell culture medium was added to each well. The cells were allowed to grow for 24 h before TTFields application started. After 72 h the cells in collagen I gels were fixed with 4% formaldehyde in PBS for 30–45 min at room temperature. Actin filaments were stained with phalloidin-iFluor^TM^ 488 (AAT Bioquest Inc., Sunnyvale, USA) in 0.3% triton X-100 in PBS for 1–2 h at room temperature. Nuclei were counterstained with 2 µM 4′,6-diamidino-2-phenylindole (DAPI) (Sigma-Aldrich). Cells were imaged with a Nikon Eclipse TE2000-E laser scanning microscope (Nikon GmbH, Düsseldorf, Germany). Five z-stacks with a step size of 15 µm were acquired from each gel. Image analysis was performed with ImageJ (http://imagej.nih.gov/ij). After applying an auto-threshold (method: default) cell clusters were manually selected and the maximal projection area as well as the perimeter and the ferret’s diameter of each cell cluster were calculated. Averages from five independent biological replicates were calculated.

**Table 4:** Lumped element values of the electromagnetic model for cells in telophase/cytokinesis (Fig. 4a).

*C*_*m*_	*R*_*m*_	*R*_*i*_	*R*_*cf*_	*R*_*e*_
10 pF	1200 MΩ	77 kΩ	735 kΩ	12 kΩ
